# Circular RNAs in cancer: from biomarkers to vaccine-based immunotherapy

**DOI:** 10.3389/fphar.2026.1930424

**Published:** 2026-09-29

**Authors:** Niranjan Kumar, Chainsee Saini, Rashi Soni, Bhavika Baweja, Prachi Patidar, Kritika Jangir, Prerna Vats, Ashok Kumar, Rajeev Nema

**Affiliations:** 1 Department of Biosciences, Manipal University Jaipur, Jaipur, Rajasthan, India; 2 Department of Biochemistry, All India Institute of Medical Sciences (AIIMS), Bhopal, Madhya Pradesh, India

**Keywords:** biomarkers, cancer vaccines, circular RNAs, neoantigen vaccines, precision oncology

## Abstract

Circular RNAs (circRNAs) are endogenous, covalently closed RNA molecules generated through back-splicing that exhibit exceptional stability, evolutionary conservation, tissue-specific expression and diverse regulatory functions. Compared with conventional RNA-based therapeutic platforms, such as messenger RNAs (mRNAs), small interfering RNAs (siRNAs), microRNAs (miRNAs), and long non-coding RNAs (lncRNAs), circRNAs offer enhanced resistance to exonuclease-mediated degradation, prolonged intracellular persistence, and sustained protein expression. These characteristics have positioned circRNAs as promising candidates for cancer biomarkers and next-generation therapeutic platforms. Recent evidence indicates that they regulate multiple biological processes associated with tumour initiation and progression, including transcriptional regulation, miRNA sequestration, RNA-binding protein modulation, alternative splicing, cap-independent translation, immune signalling, and therapeutic resistance. Through interactions with innate and adaptive immune pathways, circRNAs regulate antigen presentation, cytokine production, immune-cell activation and tumour immune evasion, highlighting their importance in cancer immunotherapy. This review summarizes recent advances in circRNA biogenesis and their roles in cancer progression, therapeutic resistance, and immune regulation, with particular emphasis on engineered circRNAs as platforms for cancer vaccines and precision immunotherapy. We discuss advances in circRNA vaccine design, delivery, neoantigen targeting, biomarkers, and clinical translation, while highlighting key challenges including manufacturing, immunogenicity, tumor heterogeneity, regulatory requirements, and limited clinical validation. Overall, circRNAs represent a promising and versatile platform for cancer immunotherapy, with continued advances in RNA engineering, personalized neoantigen design, targeted delivery, and clinical evaluation expected to determine their potential for translation into effective and durable cancer treatments.

## Highlights

- circRNAs are stable RNA platforms for next-generation cancer vaccines.
- Engineered circRNAs enable sustained protein expression and personalized immunotherapy.
- This review outlines circRNA vaccine design, production, and delivery strategies.
- Preclinical studies, clinical progress, and translational challenges are comprehensively discussed.
- circRNAs modulate innate and adaptive immune responses, supporting their potential in cancer immunotherapy.
- circRNAs contribute to chemotherapy, radiotherapy, targeted therapy, and immunotherapy resistance in cancer.

## 1 Introduction

Cancer continues to be a leading cause of death globally, despite significant progress in treatment modalities such as surgery, chemotherapy, and immunotherapy (Kaur et al., 2023). While innovative approaches like immune checkpoint inhibitors and CAR-T cell therapy have improved outcomes for some patients, their overall efficacy remains limited by intratumoral heterogeneity and therapeutic resistance (Yu et al., 2023). These challenges underscore the necessity for new therapeutic strategies capable of inducing durable and tumor-specific immune responses. In this context, RNA therapeutics are gaining traction as a promising solution to address these issues. RNA-based therapeutics have therefore emerged as promising tools in precision medicine and cancer immunotherapy. Among the major classes of therapeutic RNAs, messenger RNAs (mRNAs), small interfering RNAs (siRNAs), microRNAs (miRNAs), long non-coding RNAs (lncRNAs), and circular RNAs (circRNAs) have attracted considerable attention because of their ability to regulate gene expression through distinct molecular mechanisms. mRNA-based therapeutics primarily function by introducing protein-coding transcripts into cells, enabling transient expression of therapeutic proteins and vaccine antigens. Despite their clinical success, particularly during the COVID-19 pandemic, linear mRNAs are inherently unstable and highly susceptible to exonuclease-mediated degradation, resulting in relatively short intracellular half-lives and stringent storage requirements (Pardi et al., 2018; Sahin et al., 2014). In contrast, siRNAs silence gene expression through RNA interference by promoting the degradation of complementary mRNAs, however, their clinical application can be limited by delivery barriers, off-target effects, and transient gene silencing (Setten et al., 2019). Similarly, miRNAs regulate multiple target genes simultaneously and play essential roles in cellular homeostasis and tumor biology; however, their pleiotropic nature complicates therapeutic development because alterations in a single miRNA can affect numerous signalling pathways (Rupaimoole and Slack, 2017). lncRNAs have also emerged as important regulators of chromatin remodelling, transcriptional regulation, and post-transcriptional processing, but their large size, structural complexity, and incomplete functional characterization continue to hinder their therapeutic translation (Statello et al., 2021).

Compared with these RNA-based platforms, circRNAs possess several unique structural and functional advantages that make them attractive candidates for cancer therapeutics and vaccine development. Generated through back-splicing, circRNAs form covalently closed RNA molecules lacking 5′ and 3′ termini, which confers resistance to exonuclease-mediated degradation and contributes to their enhanced stability (Jeck et al., 2013; Memczak et al., 2013).

Their persistence within cells can support sustained protein production through cap-independent translation mediated by internal ribosome entry sites (IRESs) and N6-methyladenosine (m6A)-dependent mechanisms (Chen R. et al., 2023; Wesselhoeft et al., 2018). Unlike DNA-based therapeutics, circRNAs do not integrate into the host genome, thereby reducing the risk of insertional mutagenesis. In addition to regulating gene expression through interactions with miRNAs, RNA-binding proteins (RBPs), and other cellular components, engineered circRNAs can serve as protein-expression and immunostimulatory molecules capable of influencing innate and adaptive immune responses (Chen X. et al., 2019). These properties provide a rationale for their development as platforms for cancer vaccines, personalized immunotherapy, and precision oncology.

The growing interest in circRNAs has also expanded their relevance beyond therapeutic RNA delivery. CircRNAs were initially regarded as non-functional products of RNA splicing but are now recognized as important regulators of transcription, RNA stability, alternative splicing, miRNA activity, and, in some cases, protein translation (Fischer and Leung, 2017; Huang et al., 2022; Jiang et al., 2021; Kargar and Babakhanzadeh, 2026; Lu et al., 2021; Zhou et al., 2020). Their dysregulation has been associated with tumor initiation, progression, metastasis, immune evasion, and treatment resistance, underscoring their relevance in cancer biology (Conn et al., 2024). Accordingly, circRNAs are being investigated as diagnostic and prognostic biomarkers, therapeutic targets, and modulators of tumor immunity. Their stability also enables their detection in biological specimens, including plasma, saliva, urine, and exosomes (Pisignano et al., 2023). These features, together with advances in synthetic biology, circularization, translational optimization, and lipid nanoparticle-based delivery, have accelerated interest in engineered circRNAs for therapeutic and vaccine applications. Promising preclinical findings indicate that engineered circRNA vaccines elicit strong antigen-specific immune responses, enhance immunological memory, and inhibit tumor growth across various cancer models (Zhang et al., 2025b). The integration of next-generation sequencing, computational biology, and precision oncology has further enabled the identification of patient-specific neoantigens, leading to personalized circRNA vaccines aligned with individual tumor profiles. Despite the progress, challenges such as scalable manufacturing, purification, delivery efficiency, quality control, immunogenicity, regulatory approval, and extensive clinical validation persist. This review seeks to address a gap in the existing literature by emphasizing the relationship between circRNA biology, tumor immunology, vaccine engineering, computational design, and clinical applications, while highlighting current limitations and future directions for circRNA-based cancer vaccines in the field of precision oncology.

## 2 circRNA biogenesis and classification

A comprehensive understanding of circRNA biogenesis is essential for appreciating their biological functions and therapeutic potential. Unlike canonical linear RNA processing, circRNAs are generated through a non-canonical splicing mechanism known as back-splicing, in which a downstream 5′ splice donor is covalently linked to an upstream 3′ splice acceptor, resulting in the formation of a closed-loop RNA molecule lacking both a 5′ cap and a 3′ poly(A) tail (Jeck et al., 2013; Kristensen et al., 2019). This distinctive circular structure confers remarkable resistance to exonuclease-mediated degradation, thereby providing circRNAs with substantially greater stability than their linear counterparts (Memczak et al., 2013).

Several molecular mechanisms contribute to circRNA biogenesis. One of the most common mechanisms involves intron pairing-driven circularization, in which complementary sequences within flanking introns, particularly repetitive elements such as Alu sequences, bring splice sites into proximity and facilitate back-splicing (Jeck et al., 2013). Another mechanism is RBP-mediated circularization, in which RBPs such as Quaking (QKI), FUS, Muscleblind (MBL), and heterogeneous nuclear ribonucleoproteins (hnRNPs) interact with intronic regions and promote circRNA formation (Ashwal-Fluss et al., 2014; Conn et al., 2015). In addition, exon-skipping events may generate lariat intermediates that subsequently undergo circularization to produce mature circRNAs (Barrett et al., 2015). These regulatory mechanisms indicate that circRNA biogenesis is a tightly controlled process influenced by both cis-acting sequence elements and trans-acting regulatory proteins.

Based on their origin and structural composition, circRNAs are generally classified into three major categories: exonic circRNAs (ecircRNAs), circular intronic RNAs (ciRNAs), and exon-intron circRNAs (EIciRNAs) (Li Z. et al., 2015). The major mechanisms of circRNA biogenesis and their classification are summarized in Figure 1. ecircRNAs represent the most abundant subclass and are generated exclusively from exonic sequences. These molecules are predominantly localized in the cytoplasm, where they regulate gene expression through diverse mechanisms, including miRNA sequestration, interaction with RBPs, modulation of mRNA stability, and, in certain cases, protein translation (Hansen et al., 2013; Pamudurti et al., 2017).

**FIGURE 1:**
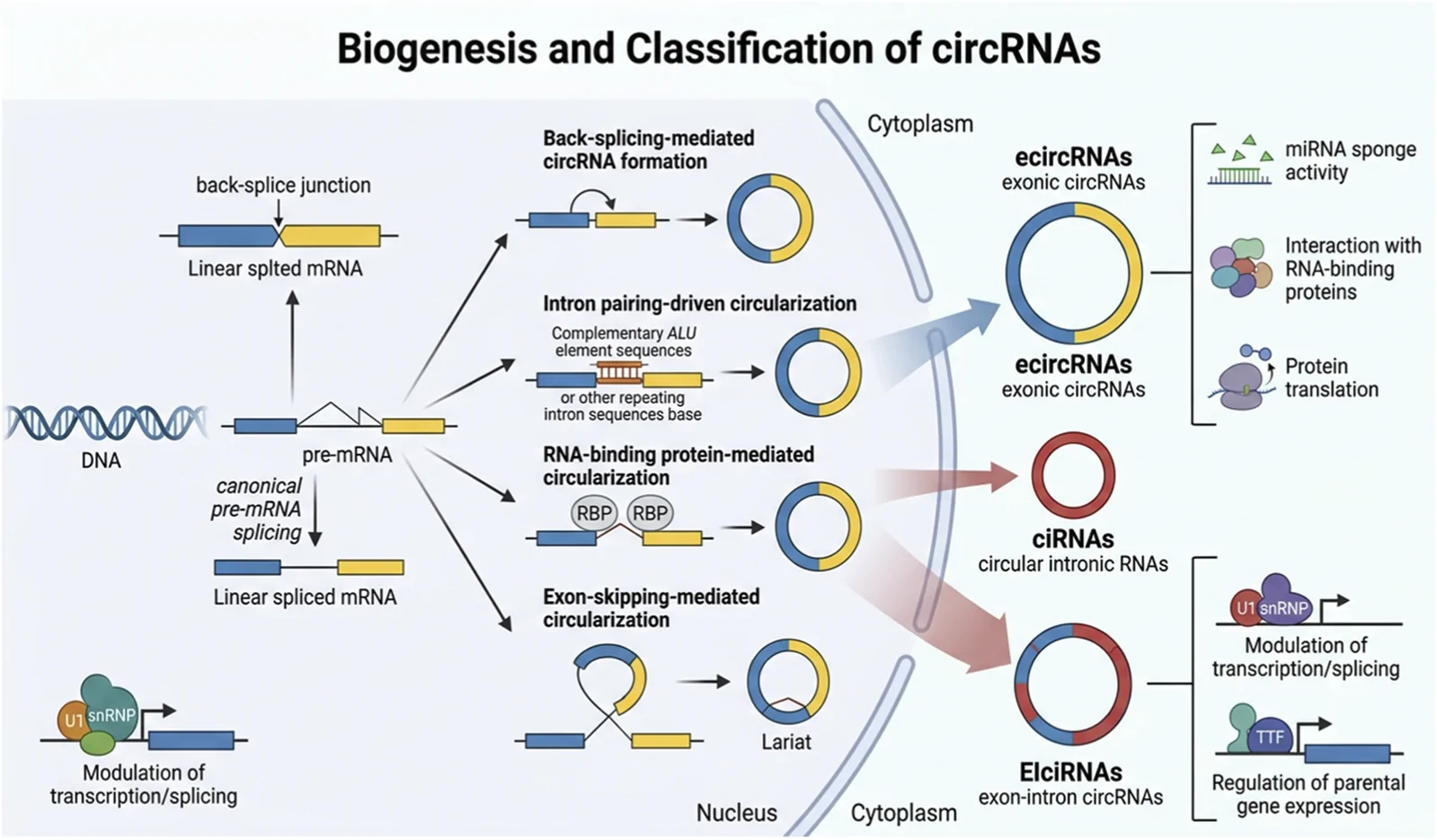
Biogenesis and classification of circular RNAs (circRNAs). CircRNAs are generated from precursor messenger RNA (pre-mRNA) through back-splicing, a non-canonical splicing mechanism that covalently joins a downstream 5′ splice donor to an upstream 3′ splice acceptor, resulting in a closed circular structure. CircRNA formation occurs through multiple mechanisms, including back-splicing-mediated circularization, intron pairing-driven circularization, RNA-binding protein (RBP)-mediated circularization, and exon-skipping-mediated circularization. Based on their composition, circRNAs are classified into exonic circRNAs (ecircRNAs), circular intronic RNAs (ciRNAs), and exon-intron circRNAs (EIciRNAs). EcircRNAs are predominantly localized in the cytoplasm and regulate gene expression through microRNA sponging, interactions with RNA-binding proteins, and protein translation. In contrast, ciRNAs and EIciRNAs are mainly localized in the nucleus, where they participate in transcriptional regulation, alternative splicing, and the modulation of parental gene expression. Abbreviations: circRNA, circular RNA; ecircRNA, exonic circular RNA; ciRNA, circular intronic RNA; EIciRNA, exon-intron circular RNA; RBP, RNA-binding protein; ALU, Arthrobacter luteus repetitive element; U1 snRNP, U1 small nuclear ribonucleoprotein; TTF, transcription factor.

In contrast, ciRNAs originate entirely from intronic sequences that escape the canonical debranching process during pre-mRNA splicing. Unlike ecircRNAs, ciRNAs are primarily localized within the nucleus, where they regulate the transcription of their parental genes by interacting with RNA polymerase II and transcriptional regulatory complexes (Zhang et al., 2013). EIciRNAs contain both exonic and intronic sequences and similarly exhibit predominantly nuclear localization. EIciRNAs contribute to transcriptional regulation by interacting with the U1 small nuclear ribonucleoprotein (U1 snRNP) complex and promoting the expression of their corresponding host genes (Li F. et al., 2015). Beyond this classical classification, recent studies have identified additional circRNA subtypes, including tRNA intronic circRNAs (tricRNAs) and mitochondria-derived circRNAs (mecciRNAs), further highlighting the structural and functional diversity of these molecules (Litke and Jaffrey, 2019; Liu et al., 2020). The diverse origins and cellular distributions of circRNAs contribute to their wide range of biological activities, including the regulation of transcription, RNA processing, protein translation, immune responses, and tumor progression. Importantly, these characteristics also provide the molecular foundation for the development of engineered circRNAs as diagnostic biomarkers, therapeutic targets, and next-generation vaccine platforms.

## 3 Role of circRNAs in cancer progression and therapeutic resistance

circRNAs are increasingly recognized as important regulators in cancer biology, impacting major aspects of tumor progression such as uncontrolled proliferation, invasion, metastasis, immune evasion, and therapeutic resistance (Ma et al., 2020). Once thought to be mere by-products of splicing, circRNAs are now acknowledged for their biological activity, participating in gene expression regulation via mechanisms like miRNA sequestration and interaction with RBPs (Fischer and Leung, 2017; Liu and Chen, 2022; Verduci et al., 2021). Their stability and tissue-specific expression allow for long-lasting effects within tumors. Their dysregulated expression has been implicated in multiple hallmarks of cancer, including proliferation, apoptosis, epithelial–mesenchymal transition (EMT), metastasis, angiogenesis, and therapeutic resistance (Hussen et al., 2025) as illustrated in Figure 2. Dysregulated circRNA levels have been implicated in cancer initiation and progression by disrupting oncogenic signaling pathways. Oncogenic circRNAs can trigger EMT, facilitating tumor cell migration and metastasis, while tumor-suppressive circRNAs counteract these processes (Shang et al., 2019). Furthermore, circRNAs play role in angiogenesis, metabolic reprogramming, and the maintenance of cancer stem cells, bolstering tumor growth in challenging environments. A notable example is circHIPK3, which is upregulated in various cancers and promotes tumor proliferation, migration, and angiogenesis by sequestering several tumor-suppressive miRNAs (Fu and Sun, 2021; Zhang et al., 2020).

**FIGURE 2:**
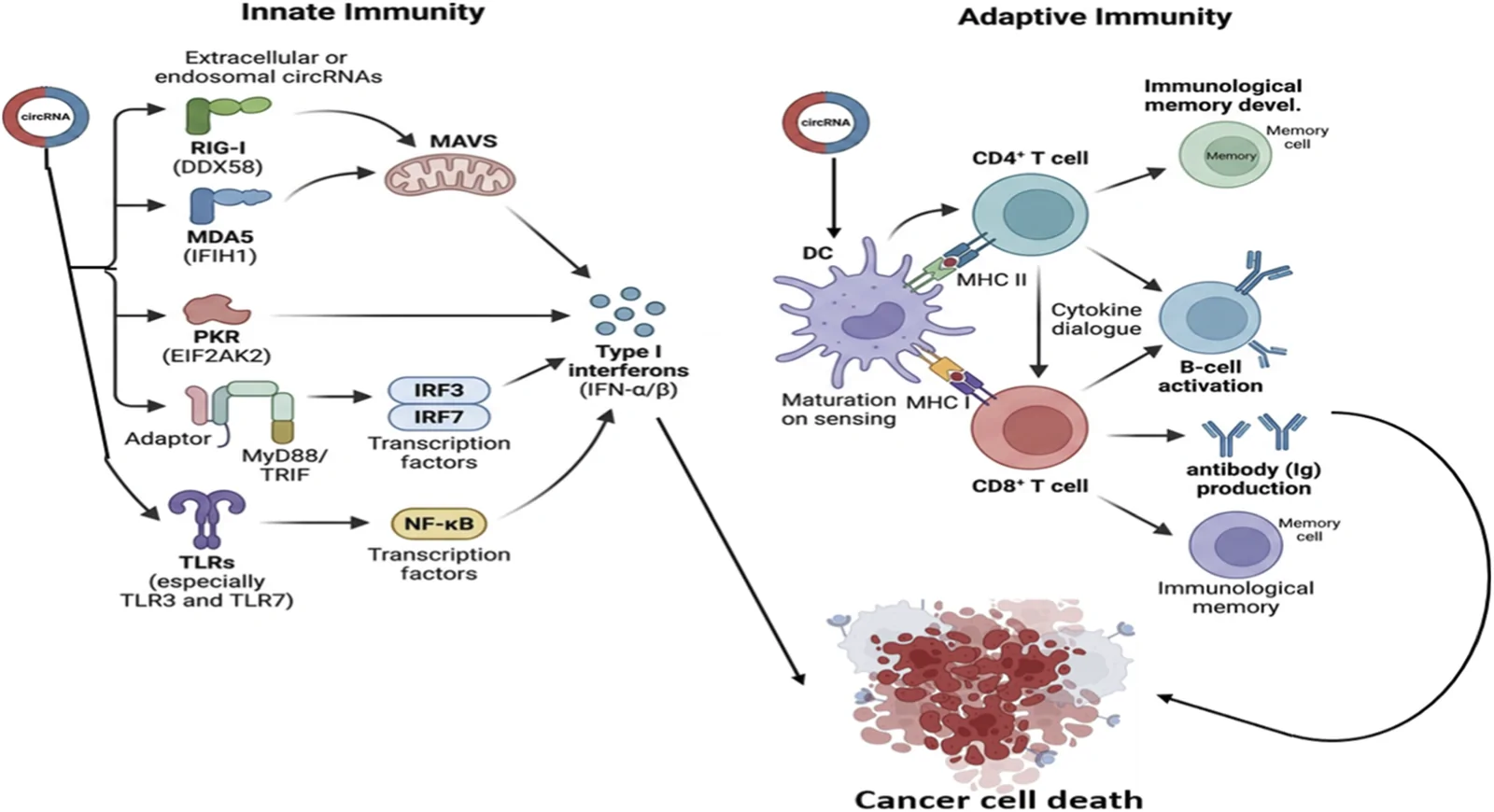
Immunological mechanism of circRNA-based cancer vaccines. Following formulation and encapsulation within delivery vehicles such as lipid nanoparticles (LNPs), circRNA vaccines are delivered to dendritic cells (DCs). Internalized circRNAs undergo cap-independent translation, producing tumor-associated or neoantigens. These antigens are processed and presented through major histocompatibility complex (MHC) molecules, leading to activation of antigen-specific CD8^+^ cytotoxic T cells, CD4^+^ helper T cells, and B-cell-mediated immune responses. Activated immune effector cells subsequently recognize and eliminate tumor cells through cytokine secretion and cytotoxic activity. The prolonged antigen expression characteristic of circRNAs further supports the generation of memory T and B cells, providing durable antitumor immunity and protection against tumor recurrence.

Likewise, particularly circPVT1 and circGFRA1, play significant roles in cancer progression and therapeutic resistance. CircPVT1, originating from the oncogenic PVT1 locus, is linked to increased cell proliferation, apoptosis inhibition, and unfavorable clinical outcomes in various cancers, including gastric, lung, and colorectal (Ghafouri-Fard et al., 2021; Ma et al., 2026; Palcau et al., 2022; Qin et al., 2019). Likewise, circGFRA1 is implicated in breast cancer advancement by affecting the miR-34a/GFRA1 signaling pathway, thus enhancing tumor growth and metastasis (Lv et al., 2021). In contrast, circRNAs like circITCH act as tumor suppressors by negatively regulating the Wnt/β-catenin pathway, leading to reduced proliferation and invasion in cancers such as colorectal and esophageal (Huang et al., 2015; Li F. et al., 2015). The emerging role of circRNAs extends beyond tumor progression; they are increasingly recognized as key players in the development of therapeutic resistance. For instance, circCRIM1 enhances chemoresistance and metastatic capabilities in nasopharyngeal carcinoma through its influence on the miR-422a/FOXQ1 signaling axis, which promotes EMT and contributes to tumor spread (Zhang Y. et al., 2024). Overall, circRNAs represent a complex layer of regulation in cancer biology, impacting both progression and treatment responses.

Similarly, circAGO2 has been shown to enhance cancer cell proliferation and metastatic behavior through its interaction with the RBP HuR, while circβ-catenin promotes activation of the Wnt/β-catenin pathway, supporting tumor growth and invasion (Chen Y. et al., 2019; Li et al., 2020). In contrast, several circRNAs exhibit tumor-suppressive functions. CircTRIM33-12 suppresses hepatocellular carcinoma progression through regulation of miR-191, whereas circHuR inhibit gastric cancer development and metastasis (Yang et al., 2019; Zhang et al., 2019). The balance between oncogenic and tumor-suppressive circRNAs, therefore, represents an important determinant of disease progression and treatment response. Additional evidence highlights the diverse roles of circRNAs in regulating malignant phenotypes across different tumor types. circFOXO3 has been reported to exert tumor-suppressive effects by interacting with cell cycle regulatory proteins and restricting cancer cell proliferation, whereas circCCDC66 promotes colorectal cancer progression by enhancing the expression of genes associated with cell survival, migration, and invasion (Li et al., 2019; Mo et al., 2022). Similarly, circRNA CDR1as (ciRS-7), one of the most extensively characterized circRNAs, functions as a potent sponge for miR-7 and contributes to tumor progression through dysregulation of multiple oncogenic pathways, including EGFR, PI3K/AKT, and RAF/MEK/ERK signaling. These observations further emphasize the context-dependent roles of circRNAs as either drivers or suppressors of tumorigenesis (Greene et al., 2017; Liu et al., 2021).

There is increasing evidence that circRNAs play a significant role in cellular sensitivity to anticancer therapies by regulating apoptosis, drug efflux, DNA damage repair, and survival signaling pathway (Cui et al., 2020; Misir et al., 2025; Mu et al., 2021). In various cancers, circRNAs act as competing endogenous RNAs (ceRNAs), sequestering miRNAs that are involved in drug response and thereby altering the expression of genes associated with drug resistance. For instance, circ_0076305 has been shown to contribute to cisplatin resistance in non-small cell lung cancer (NSCLC) by modulating the miR-296–5p/STAT3 axis (Dong et al., 2019). Other circRNAs have been associated with resistance to therapies such as paclitaxel, tamoxifen, and targeted agents. For example, circBMPR2 has been reported to affect tamoxifen sensitivity in breast cancer through regulation of the miR-553/USP4 axis (Liang Y. et al., 2019). In hepatocellular carcinoma, circRNA-SORE promotes resistance to sorafenib by preventing the degradation of oncogenic signaling proteins, thereby sustaining pro-survival pathways (Chen X. et al., 2023; Hua et al., 2025). These findings indicate that circRNA-mediated therapeutic resistance is not limited to a single treatment type but is a widespread mechanism contributing to treatment failure across various cancer types. The growing interest in circRNAs as potential therapeutic targets is a result of these findings. Researchers are exploring strategies such as siRNA, short hairpin RNA (shRNA), antisense oligonucleotides, and CRISPR-based approaches to selectively inhibit oncogenic circRNAs and restore treatment sensitivity. Depending on their molecular targets and signaling pathways, circRNAs may function either as oncogenic drivers or tumor suppressors. Representative circRNAs involved in cancer progression and therapeutic resistance are summarized in Table 1. Overall, the accumulating evidence underscores circRNAs as key regulators of cancer progression and therapeutic resistance, providing a solid rationale for utilizing these molecules in next-generation cancer immunotherapy and vaccine development.

**TABLE 1:** Functional roles of circRNAs in cancer progression and therapeutic resistance.

circRNA	Cancer type	Biological effect	References
circHIPK3	Multiple cancers	Promotes proliferation, angiogenesis, migration	Chen et al. (2018); Wen et al. (2022); Yao et al. (2022)
circPVT1	Gastric, Lung, CRC	Cell proliferation, anti-apoptosis	Tolomeo et al. (2023)
circGFRA1	Breast Cancer	Tumor growth and metastasis	Bazhabayi et al. (2021)
circITCH	CRC, ESCC	Tumor suppression	Huang et al. (2015)
circAGO2	Multiple cancers	Metastasis and proliferation	Chen Y. et al. (2019)
circβ-catenin	Various cancers	Tumor invasion	Liang W. et al. (2019)
circTRIM33-12	HCC	Tumor suppression	Zhang et al. (2019)
circFOXO3	Multiple cancers	Anti-proliferative effect	Rao et al. (2021)
CDR1as (ciRS-7)	Multiple cancers	Activates oncogenic signaling	Chen et al. (2021)

## 4 Why circRNAs are attractive vaccine platforms

The rapid advancement of RNA-based therapeutics has transformed vaccine development, particularly following the global success of mRNA vaccines. Despite their remarkable clinical impact, linear mRNA molecules possess inherent limitations, including susceptibility to exonuclease-mediated degradation, relatively short intracellular half-lives, and demanding storage requirements (Sadaqa et al., 2026). circRNAs have emerged as an attractive alternative platform capable of overcoming many of these challenges. Structurally, circRNAs are characterized by a covalently closed loop lacking both 5′ and 3′ termini, rendering them naturally resistant to exonuclease activity (Saw and Song, 2025). This unique architecture confers substantially greater stability than linear RNA molecules and allows prolonged persistence within cells, leading to sustained protein expression (Bahrulolum et al., 2026). Experimental studies have demonstrated that circRNAs can maintain antigen production for extended periods compared with conventional mRNA constructs, making them particularly appealing for vaccine applications where durable antigen expression is desirable.

Another major advantage of circRNA vaccines lies in their ability to support efficient cap-independent translation. Unlike conventional mRNA molecules that rely on a 5′ cap structure for ribosome recruitment, circRNAs can initiate translation through IRESs and (m6A)-mediated mechanisms (Hwang and Kim, 2024). These alternative translational pathways permit robust protein synthesis despite the absence of canonical mRNA features. Advances in synthetic biology have further improved translational efficiency through codon optimization, sequence engineering, and incorporation of translation-enhancing elements, enabling engineered circRNAs to produce therapeutically relevant antigen levels (Qu et al., 2022; Wesselhoeft et al., 2018). The key differences between conventional mRNA and circRNA vaccine platforms in terms of stability, translation, manufacturing, and clinical maturity are summarized in Table 2. Importantly, circRNAs do not integrate into the host genome, reducing concerns associated with genomic insertion and enhancing their safety profile (Qu et al., 2022). Their cell-free manufacturing process also facilitates rapid vaccine production and adaptation to emerging therapeutic targets (Wesselhoeft et al., 2019).

**TABLE 2:** Preclinical and clinical progress of circRNA-based cancer vaccines.

circRNA-based vaccine	Target antigen/encoded cargo	Cancer type/model	Delivery system	Therapeutic strategy	Development stage	References
HCC circRNA neoantigen vaccine	Patient/tumor-derived HCC neoantigens	Hepatocellular carcinoma	LNPs	Personalized neoantigen vaccination	Preclinical	Wang F. et al. (2024)
CircFAM53B-219aa DC vaccine	Cryptic peptide FAM53B-219aa	HER2-negative advanced breast cancer	Autologous dendritic cells loaded with circRNA	DC-based therapeutic vaccination ± camrelizumab	Phase I	Maeng et al. (2021)
circRNA NY-ESO-1 vaccine	NY-ESO-1 cancer-testis antigen	Lung cancer models	LNPs	Therapeutic tumor vaccination ± immune checkpoint blockade	Preclinical	Chen S. et al. (2025)
LNP-circRNA E6/E7 (TI-0093)	HPV16 E6/E7 oncoproteins	HPV16-positive recurrent/metastatic solid tumors	LNPs	Therapeutic tumor vaccination	Phase I; recruiting	Therorna (2025)
HER2-targeting circRNA vaccine + CAR-T	HER2 fusion protein/anti-HER2 CAR	Colorectal and breast cancer models	LNPs	CircRNA vaccination combined with CAR-T therapy	Preclinical	​
circRNA vaccine + TCR-T	OVA	Colorectal cancer/melanoma models	LNPs	CircRNA vaccination combined with TCR-engineered T cells	Preclinical	​
SARS-CoV-2 circRNA Vaccine	RBD antigen	​	​	​	Preclinical	Qu et al. (2022)

In addition to their favorable stability and translational characteristics, circRNAs possess unique immunological properties that distinguish them from other vaccine platforms. Synthetic circRNAs can activate innate immune pathways through recognition by pattern recognition receptors, thereby providing intrinsic adjuvant activity capable of enhancing vaccine-induced immune responses (Liu et al., 2019; Niu et al., 2023). This self-adjuvant potential may reduce dependence on external adjuvants while promoting efficient activation of antigen-presenting cells and downstream adaptive immunity (Kowalczyk et al., 2016). Furthermore, the prolonged antigen expression achieved by circRNA vaccines can support sustained stimulation of T cells and facilitate the generation of durable immunological memory (Bu et al., 2025). Compared with DNA vaccines, circRNAs eliminate concerns regarding genomic integration, while compared with conventional mRNA vaccines they offer improved stability, longer protein expression, and potentially simplified storage requirements (Zhang Z. et al., 2024). Together, these characteristics position circRNAs as highly promising next-generation vaccine platforms with significant potential for cancer immunotherapy and personalized vaccine development.

## 5 circRNA-based vaccines for cancer: an immunological perspective

The therapeutic potential of circRNA-based cancer vaccines lies in their ability to generate coordinated innate and adaptive immune responses capable of recognizing and eliminating malignant cells. Following cellular uptake, engineered circRNAs are translated into tumor-associated antigens or neoantigens through cap-independent mechanisms such as IRES- and m6A-mediated translation (Ding et al., 2026; Savguira et al., 2026). These antigens are subsequently processed and presented by major histocompatibility complex (MHC) molecules, allowing immune cells to distinguish tumor cells from healthy tissues (Balakrishnan et al., 2025). The resulting prolonged antigen expression can support sustained immune stimulation (Chen X. et al., 2019). This extended period of antigen availability is particularly advantageous in cancer immunotherapy, where durable immune activation is often required to overcome tumor-induced immune suppression. The initiation of vaccine-induced immunity depends largely on the activation of antigen-presenting cells, particularly dendritic cells (DCs), which serve as a critical link between innate and adaptive immunity (Garg et al., 2012). Emerging evidence suggests that circRNAs are not merely passive carriers of genetic information but can actively participate in immune activation. In a landmark study by Amaya and colleagues, a circular RNA vaccine encoding the model antigen ovalbumin (circOVA) triggered activation of DCs, monocytes, and macrophages within draining lymph nodes, creating an immunological environment favorable for T-cell priming. This innate immune activation was accompanied by robust antigen presentation and the induction of potent adaptive immune responses, highlighting the dual role of circRNAs as both antigen-expression platforms and immunostimulatory molecules (Amaya et al., 2023). The immunological mechanism underlying circRNA-based cancer vaccines involves a series of coordinated events, including vaccine delivery, intracellular antigen production, antigen presentation, activation of adaptive immune responses, tumor elimination, and the establishment of long-term immunological memory (Huang et al., 2026). These processes are summarized in Figure 3.

**FIGURE 3:**
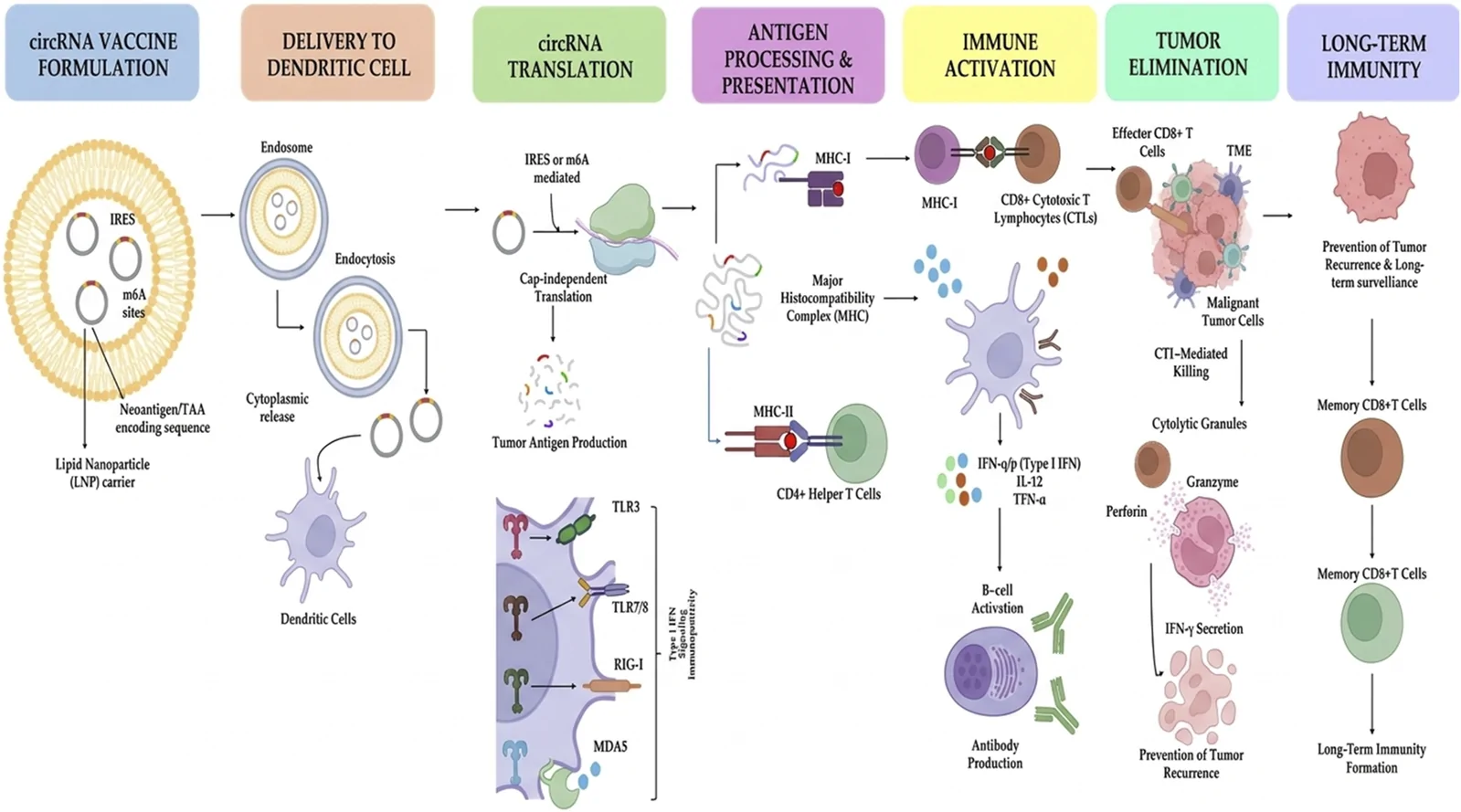
Schematic illustration of the role of circRNAs in regulating innate and adaptive immune responses: In innate immunity, circRNAs activate immune sensors, including RIG-I, MDA5, PKR, and TLRs, leading to the activation of MAVS, IRF3/IRF7, and NF-κB signalling pathways and the subsequent production of type I interferons (IFN-α/β). In adaptive immunity, circRNAs promote dendritic cell maturation, antigen presentation, T-cell activation, B-cell-mediated antibody production, and the development of immunological memory. These coordinated immune responses enhance the recognition and elimination of cancer cells, highlighting the therapeutic potential of circRNAs in cancer immunotherapy.

A major objective of therapeutic cancer vaccines is the generation of strong cytotoxic CD8^+^ T-cell responses capable of directly killing tumor cells. Preclinical studies have demonstrated that circRNA vaccination can efficiently achieve this goal. The circOVA vaccine induced vigorous antigen-specific CD8^+^ T-cell expansion together with enhanced production of effector cytokines such as interferon-γ, ultimately translating into significant tumor growth inhibition in murine models (Rapaka et al., 2021). Sustained antigen expression from circRNAs may help maintain prolonged T-cell stimulation, thereby improving both the magnitude and durability of antitumor immune responses.

Recent discoveries have further expanded the immunological relevance of circRNAs by revealing that some endogenous tumor-derived circRNAs can themselves serve as sources of immunogenic antigens. For example, circFAM53B was found to encode cryptic peptides capable of being recognized by T cells, thereby contributing to antitumor immune surveillance (Huang D. et al., 2024). This observation challenges the traditional view of circRNAs as non-coding molecules and suggests that tumor-specific circRNA-derived peptides may represent an untapped reservoir of vaccine targets. The ability to identify and exploit such naturally occurring circRNA-derived antigens could facilitate the development of highly personalized immunotherapies tailored to the molecular characteristics of individual tumors. In addition to generating immediate effector responses, effective cancer vaccines must establish long-term immunological memory capable of preventing tumor recurrence. Such findings suggest that circRNA vaccines may not only suppress established tumors but could also provide prolonged protection against disease relapse. The versatility of the circRNA platform is particularly evident in the context of personalized cancer immunotherapy. Advances in next-generation sequencing have enabled the identification of patient-specific neoantigens, creating opportunities for individualized vaccine design. Supporting this concept, a recently developed circRNA-based neoantigen vaccine for hepatocellular carcinoma enhanced dendritic-cell maturation, promoted antigen-specific T-cell activation, and significantly suppressed tumor progression in preclinical models (Wang F. et al., 2024). These findings demonstrate the feasibility of combining precision oncology with circRNA technology to generate tailored immune responses against unique tumor antigens.

Although most cancer-focused circRNA vaccine studies remain at the preclinical stage, additional support for the immunogenicity of this platform comes from infectious disease research. circRNA vaccines encoding the receptor-binding domain (RBD) of SARS-CoV-2 have been shown to induce strong neutralizing antibody responses together with robust Th1-biased cellular immunity (Qu et al., 2022). While these studies were not designed for cancer treatment, they provide important proof-of-principle evidence that circRNA constructs can elicit broad and durable immune responses *in vivo*. Although these findings are outside oncology, they provide complementary evidence supporting the broader immunological potential of circRNA vaccine technology.

## 6 circRNAs in immune regulation

Beyond their well-established roles in gene regulation and tumor progression, circRNAs have emerged as important modulators of the immune system. Recent evidence suggests that circRNAs actively participate in both innate and adaptive immune signaling by regulating immune cell activation, cytokine production, antigen presentation, and immune surveillance. These immunomodulatory properties have attracted considerable attention, particularly in cancer immunotherapy, where the ability to simultaneously regulate multiple immune pathways may enhance therapeutic efficacy (Chen X. et al., 2019; Liu et al., 2019).

### 6.1 circRNAs and innate immune signaling

The innate immune system serves as the first line of defense against pathogens and malignant cells by recognizing conserved molecular patterns through pattern-recognition receptors (PRRs). Synthetic and endogenous circRNAs can interact with several PRRs, including retinoic acid-inducible gene-I (RIG-I), melanoma differentiation-associated protein 5 (MDA5), protein kinase R (PKR), and Toll-like receptors (TLRs), thereby influencing downstream inflammatory signaling pathways (Chen X. et al., 2019). RIG-I and MDA5 are cytoplasmic RNA sensors that recognize foreign RNA molecules and initiate antiviral immune responses. Recognition of immunogenic circRNAs by these receptors activates mitochondrial antiviral signaling protein (MAVS), which subsequently triggers the phosphorylation of interferon regulatory factors 3 and 7 (IRF3 and IRF7) and the activation of nuclear factor-κB (NF-κB). These signaling events ultimately stimulate the production of type I interferons and pro-inflammatory cytokines that promote innate immune activation and shape subsequent adaptive immune responses (Liu et al., 2019). The major signaling pathways through which circRNAs influence innate and adaptive immune responses are illustrated in Figure 4.

**FIGURE 4:**
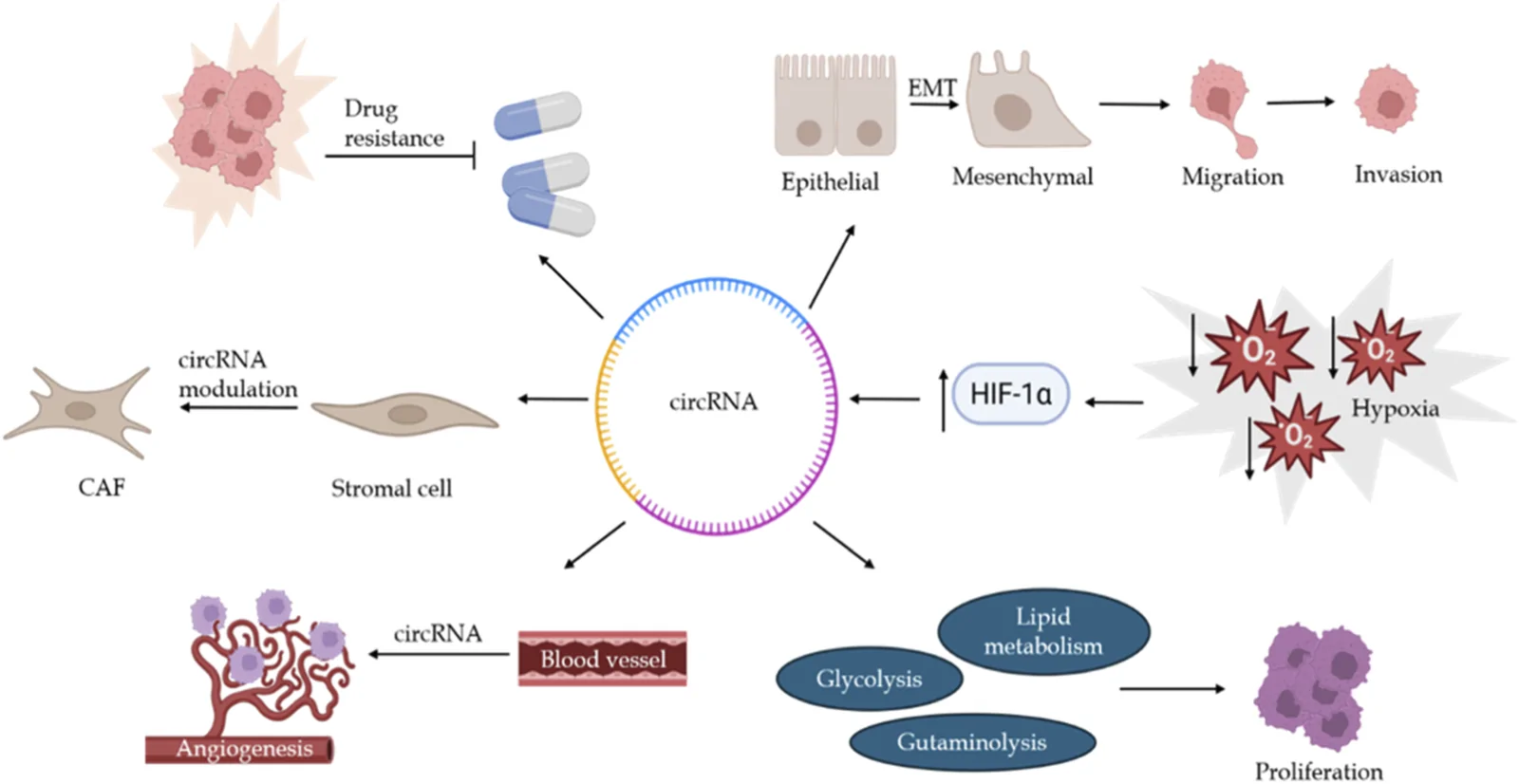
Multifaceted roles of circRNAs in cancer progression and therapeutic resistance. CircRNAs regulate multiple hallmarks of cancer through diverse molecular mechanisms. Dysregulated circRNA expression contributes to uncontrolled cell proliferation, resistance to apoptosis, epithelial–mesenchymal transition (EMT), invasion, metastasis, angiogenesis, metabolic reprogramming, hypoxia adaptation through HIF-1α signaling, and therapeutic resistance. By interacting with microRNAs, RNA-binding proteins, and key oncogenic pathways, circRNAs modulate tumor growth, remodeling of the tumor microenvironment, and cancer progression, highlighting their potential as biomarkers and therapeutic targets.

Another important innate immune regulator influenced by circRNAs is PKR, a double-stranded RNA-dependent protein kinase involved in antiviral defense and inflammatory responses. Under physiological conditions, endogenous circRNAs can bind PKR and maintain it in an inactive state. During viral infection or cellular stress, circRNA degradation releases PKR, resulting in its activation and the subsequent induction of inflammatory signaling pathways (Liu et al., 2019). These findings suggest that circRNAs function not only as RNA molecules but also as regulators of intracellular immune surveillance mechanisms. circRNAs also influence the activity of macrophages, natural killer (NK) cells, and dendritic cells (DCs). Several tumor-derived circRNAs regulate macrophage polarization by promoting either the antitumor M1 phenotype or the immunosuppressive M2 phenotype, thereby influencing the composition of the tumor microenvironment. Similarly, circRNAs have been shown to regulate NK-cell cytotoxicity and cytokine production, affecting the immune system’s ability to recognize and eliminate tumor cells (Chen X. et al., 2019).

However, the immunostimulatory properties of circRNAs must be balanced against their translational efficiency. Excessive activation of innate immune sensors can compromise circRNA translation and therapeutic performance. Consequently, current strategies aim to minimize unintended immune activation by improving circRNA purity and structural fidelity, particularly through the removal of residual linear RNA, double-stranded RNA (dsRNA), and incompletely circularized products that can stimulate innate immune sensors (Zhao et al., 2025). Sequence and structural optimization of circRNAs is also being investigated to reduce recognition by sensors such as RIG-I and MDA5 while maintaining efficient translation (Niu et al., 2023). Chemical modification of circRNAs, including m6A incorporation, represents another potential approach; however, its effects on circRNA immunogenicity and circularization remain context-dependent and require further investigation.

### 6.2 circRNAs and adaptive immune signaling

Although innate immunity provides immediate protection, adaptive immunity is responsible for generating highly specific and long-lasting antitumor responses. Emerging evidence indicates that circRNAs play essential roles in regulating adaptive immune signaling through their effects on antigen presentation, T-cell activation, B-cell responses, and immunological memory. DCs represent a critical link between innate and adaptive immunity because they capture antigens and present them to T lymphocytes through major histocompatibility complex (MHC) molecules. Through their effects on antigen-presenting cells, circRNAs can influence the efficiency of T-cell priming and subsequent adaptive immune activation.

Engineered circRNAs have demonstrated the ability to stimulate dendritic-cell maturation and enhance antigen presentation, thereby improving T-cell priming and activation (Amaya et al., 2023). The resulting antigen presentation can promote the activation of CD8^+^ cytotoxic T cells and CD4^+^ helper T cells, which contribute to antitumor immunity through complementary mechanisms. CD8^+^ T cells directly recognize and eliminate malignant cells, whereas CD4^+^ T cells provide essential support for cytotoxic T-cell expansion and the maintenance of sustained immune responses. In the context of circRNA-based vaccination, these responses have been associated with increased production of effector cytokines, including interferon-γ (IFN-γ), and suppression of tumor growth (Amaya et al., 2023). Recent evidence also suggests that certain endogenous tumor-derived circRNAs can encode immunogenic peptides that serve as novel antigens recognized by T cells. For example, circFAM53B-derived peptides have been shown to trigger T-cell-mediated antitumor responses, suggesting that circRNA-derived neoantigens may represent an untapped source of therapeutic targets (Li et al., 2024).

In addition to T-cell-mediated immunity, circRNAs influence B-cell activation and antibody production, further contributing to adaptive immune regulation (Hsu et al., 2025). Their effects on antigen presentation and immune-cell activation may also contribute to the establishment of immunological memory, although the mechanisms governing durable memory responses remain incompletely understood. Further investigation is therefore required to define how circRNA-mediated regulation of adaptive immunity influences the magnitude, specificity, and persistence of antitumor responses. An overview of the interconnected innate and adaptive immune pathways regulated by circRNAs is presented in Figure 4.

### 6.3 circRNAs in tumor immune evasion

CircRNAs also participate in mechanisms of tumor immune escape. Several oncogenic circRNAs regulate the expression of immune checkpoint molecules, including programmed death-ligand 1 (PD-L1), thereby suppressing T-cell activity and facilitating immune evasion. Furthermore, circRNAs can remodel the tumor microenvironment by regulating inflammatory cytokines, angiogenesis, macrophage polarization, and immune-cell infiltration. These immunosuppressive effects promote tumor progression and reduce the effectiveness of antitumor immune responses (Conn et al., 2024).

Collectively, circRNAs influence tumor immunity through interconnected innate and adaptive immune pathways, ranging from pattern-recognition receptor signaling and cytokine production to antigen presentation, lymphocyte activation, and immune evasion (Xu et al., 2026). These interactions provide a mechanistic basis for understanding both the immunostimulatory potential and the immunosuppressive effects of circRNAs in cancer, while also highlighting the need to balance immune activation with efficient therapeutic translation. Beyond their roles in cancer biology and immune regulation, circRNAs are being explored across diverse clinical applications, including diagnostic and prognostic biomarkers, therapeutic targets, cancer immunology, antiviral applications, and vaccine development, as summarized in Figure 5.

**FIGURE 5:**
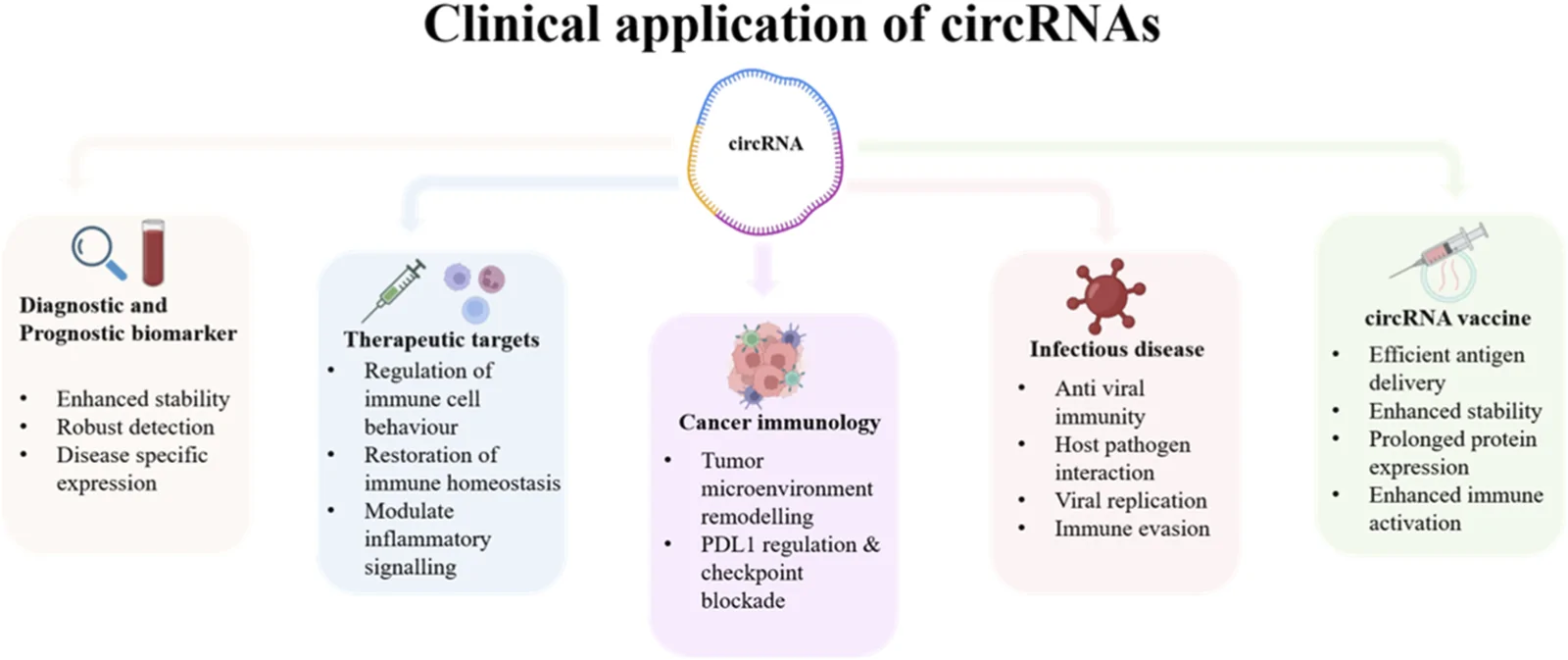
Clinical applications of circRNAs. circRNAs have emerged as versatile molecules with broad clinical relevance. Their exceptional stability and disease-specific expression profiles support their use as diagnostic and prognostic biomarkers. circRNAs also serve as therapeutic targets by regulating immune cell behavior, inflammatory signaling, and immune homeostasis. In cancer, circRNAs contribute to tumor microenvironment remodeling and immune checkpoint regulation. Furthermore, circRNAs participate in host–pathogen interactions and antiviral immunity and have recently gained attention as promising vaccine platforms due to their enhanced stability, prolonged protein expression, efficient antigen delivery, and ability to induce robust immune responses.

## 7 circRNAs and therapeutic resistance in cancer

Therapeutic resistance remains one of the most significant challenges in cancer treatment and is a major cause of disease progression, metastasis, tumor recurrence, and poor patient survival (Khan et al., 2024). Although chemotherapy, radiotherapy, targeted therapy, and immunotherapy have improved clinical outcomes, many tumors eventually develop resistance through genetic, epigenetic, and microenvironmental adaptations (Mubin et al., 2026). Emerging evidence suggests that circRNAs are important regulators of these resistance mechanisms because they influence multiple signaling pathways involved in cell survival, proliferation, apoptosis, DNA repair, immune responses, and cellular plasticity (Tang et al., 2026). The major mechanisms by which circRNAs contribute to resistance to chemotherapy, radiotherapy, targeted therapy, and immunotherapy are summarized in Figure 6.

**FIGURE 6:**
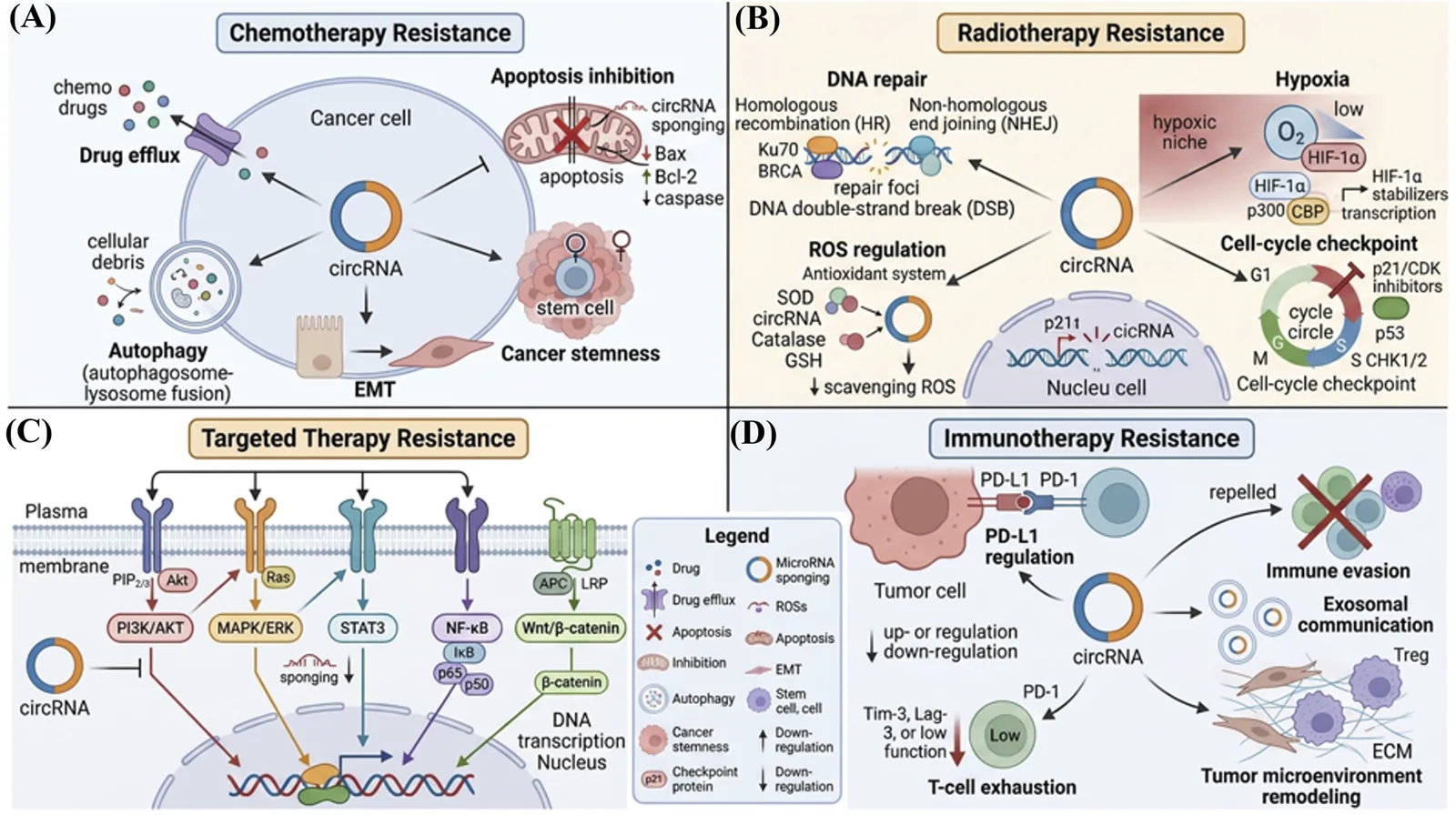
Molecular mechanisms of circRNA-mediated therapeutic resistance in cancer. CircRNAs contribute to resistance to **(A)** Chemotherapy by regulating drug efflux, apoptosis, autophagy, epithelial-mesenchymal transition (EMT), and cancer stemness. **(B)** Radiotherapy resistance is mediated through the modulation of DNA repair, reactive oxygen species (ROS) homeostasis, hypoxia-associated signaling, and cell-cycle checkpoints. **(C)** Targeted therapy resistance involves the activation of alternative oncogenic pathways, including PI3K/AKT, MAPK/ERK, STAT3, NF-κB, and Wnt/β-catenin signaling. **(D)** Immunotherapy resistance is promoted through the regulation of the PD-1/PD-L1 axis, T-cell exhaustion, exosomal communication, immune evasion, and tumor microenvironment remodelin.

### 7.1 circRNAs and chemotherapy resistance

Chemotherapy remains one of the most widely used treatment strategies for cancer. However, prolonged exposure to chemotherapeutic agents often results in the development of drug-resistant tumor cell populations (Martinez et al., 2026). CircRNAs contribute to chemotherapy resistance through several molecular mechanisms. One of the primary mechanisms involves the regulation of drug efflux transporters. ATP-binding cassette (ABC) transporters, including ABCB1, ABCC1, and ABCG2, actively export anticancer drugs from tumor cells, reducing intracellular drug concentrations and limiting therapeutic efficacy (Wu et al., 2026). CircRNAs regulate the expression of these transporters through circRNA-miRNA-mRNA regulatory networks, thereby enhancing drug resistance (Chen X. et al., 2026). CircRNAs also influence apoptotic signaling pathways. The effectiveness of many chemotherapeutic agents depends on their ability to induce programmed cell death. However, several circRNAs suppress apoptosis by increasing the expression of anti-apoptotic proteins, such as Bcl-2, while simultaneously reducing the expression of pro-apoptotic molecules, including Bax and caspases. This imbalance promotes tumor cell survival despite chemotherapy-induced cellular stress (Hashemi et al., 2026).

Another important mechanism is the regulation of autophagy, a lysosome-dependent degradation pathway that enables tumor cells to recycle damaged cellular components and adapt to unfavorable conditions. circRNAs activate autophagy-related pathways, particularly the PI3K/AKT/mTOR pathway, thereby allowing cancer cells to survive during chemotherapy (Zhang Y. et al., 2026). Furthermore, circRNAs promote EMT and maintain cancer stem cell (CSC) populations. EMT enhances cellular migration and invasiveness, whereas CSCs possess self-renewal properties and exhibit reduced sensitivity to chemotherapeutic agents (Jain et al., 2026; Zhou et al., 2026). Together, these mechanisms contribute to the development of multidrug resistance.

### 7.2 circRNAs and radiotherapy resistance

Radiotherapy eliminates tumor cells primarily by inducing DNA damage and generating reactive oxygen species (ROS) (Guo et al., 2025). However, tumor cells frequently develop adaptive responses that enable them to survive radiation-induced stress. circRNAs contribute to radioresistance by regulating DNA repair pathways, including homologous recombination (HR) and non-homologous end joining (NHEJ) (Wilson et al., 2026). These pathways repair radiation-induced DNA double-strand breaks through the activation of repair proteins such as ATM, ATR, BRCA1, RAD51, and DNA-PK (Hong and Li, 2026; P. P. Rao and Vyas, 2026). circRNAs also regulate oxidative stress responses by modulating the expression of antioxidant enzymes, including superoxide dismutase (SOD), catalase, and glutathione (GSH) (Liu Z. et al., 2026). Enhanced antioxidant activity reduces ROS accumulation and protects tumor cells from radiation-induced damage.

In addition, circRNAs regulate cell-cycle checkpoints through the modulation of proteins such as p21, p53, CHK1, CHK2, and cyclin-dependent kinases (CDKs). These proteins allow tumor cells to temporarily arrest cell-cycle progression, repair damaged DNA, and resume proliferation (Wan et al., 2026). Another important factor is hypoxia, which is commonly observed within solid tumors. CircRNAs regulate hypoxia-inducible factor-1α (HIF-1α) signaling, promoting angiogenesis, metabolic adaptation, and resistance to radiation therapy (Hu et al., 2026).

### 7.3 circRNAs and targeted therapy resistance

Targeted therapies selectively inhibit specific oncogenic molecules that drive tumor progression. Although these therapies initially demonstrate remarkable efficacy, resistance frequently develops through reactivation of downstream signaling pathways and activation of compensatory survival mechanisms. CircRNAs promote resistance to targeted therapies by regulating several oncogenic pathways, including PI3K/AKT, MAPK/ERK, STAT3, NF-κB, and Wnt/β-catenin signaling (Molavand et al., 2025). These pathways regulate cell survival, proliferation, inflammation, stemness, and cellular differentiation and can provide alternative signaling routes that sustain tumor-cell survival despite pharmacological inhibition (Aiyejuto et al., 2026).

Through their ability to function as ceRNAs, circRNAs sequester tumor-suppressive miRNAs thereby restore the expression of target genes involved in these signaling pathways. Such circRNA–miRNA–mRNA networks can reactivate oncogenic signaling and reduce the sensitivity of tumor cells to targeted agents. CircRNAs also promote metabolic reprogramming, enabling tumor cells to alter glucose utilization, lipid metabolism, and mitochondrial function in response to therapeutic stress. These metabolic adaptations provide alternative energy sources that support tumor cell survival.

### 7.4 circRNAs and immunotherapy resistance

Immunotherapy has transformed cancer treatment by restoring the immune system’s ability to recognize and eliminate malignant cells. Nevertheless, many patients fail to respond because tumors develop mechanisms that suppress antitumor immunity. circRNAs contribute to immunotherapy resistance by regulating immune checkpoint molecules, particularly PD-L1. Increased PD-L1 expression inhibits PD-1 signaling on T cells, suppressing cytotoxic immune responses and promoting immune escape. circRNAs also regulate T-cell exhaustion by influencing inhibitory receptors, including TIM-3 and LAG-3. Persistent activation of these pathways reduces T-cell proliferation and cytokine production.

Another important mechanism involves the remodeling of the TME. CircRNAs regulate macrophage polarization, cytokine production, extracellular matrix remodeling, and the recruitment of immunosuppressive cells, including regulatory T cells (Tregs) and myeloid-derived suppressor cells (MDSCs). Furthermore, exosomal circRNAs facilitate intercellular communication between tumor cells and immune cells. These extracellular vesicles transfer resistance-associated circRNAs throughout the TME, promoting immune evasion and reducing the effectiveness of immunotherapeutic agents.

## 8 Design and delivery of circRNA-based cancer vaccines

### 8.1 In silico designing of circRNA

The development of circRNA-based cancer vaccines begins with the identification of suitable antigens capable of eliciting robust and tumor-specific immune responses (Gong et al., 2025). Recent advances in next-generation sequencing technologies have enabled comprehensive characterization of tumor genomes, facilitating the discovery of somatic mutations that can serve as potential neoantigens (Efremova et al., 2017). These neoantigens are particularly attractive vaccine targets because they are absent in normal tissues and therefore less likely to induce immune tolerance (Jiang et al., 2019). Computational approaches have become indispensable in this process, allowing researchers to analyze genomic and transcriptomic datasets, predict peptide–MHC binding affinities, and prioritize antigenic epitopes with the highest immunogenic potential. Bioinformatics tools such as NetMHC, NetMHCpan, MHCflurry, and related prediction platforms are commonly employed to evaluate antigen presentation and T-cell recognition probabilities (Schaap-Johansen et al., 2021). More recently, machine learning and artificial intelligence-based algorithms have further improved the accuracy of neoantigen prediction by integrating multiple parameters including antigen processing, MHC presentation, and T-cell receptor recognition (Seth et al., 2025). Advances in RNA sequencing and bioinformatics have led to the development of comprehensive circRNA databases such as MiOncoCirc, CircBase, CircAtlas, and CSCD, facilitating the identification of cancer-specific circRNAs across numerous tumor types (Vo et al., 2019).

The importance of computational vaccine design is particularly evident in personalized cancer immunotherapy, where patient-specific mutations can be rapidly translated into customized vaccine constructs. The successful development of a circRNA-based neoantigen vaccine for hepatocellular carcinoma demonstrated the feasibility of this approach, highlighting how genomic information can be utilized to generate individualized therapeutic vaccines capable of eliciting targeted antitumor immune responses (Wang F. et al., 2024). Representative examples include the PTPN2^I383T neoantigen, which has been incorporated into an LNP-formulated circRNA vaccine for hepatocellular carcinoma, and the MC38 tumor-specific ADPGK neoantigen, which has been evaluated using a small circRNA vaccine platform. More recently, multivalent circRNA vaccines encoding melanoma-associated neoantigens, including TRP2_180–190 and human gp100_23–33, have also demonstrated the potential of circRNA platforms to target multiple tumor antigens simultaneously. Collectively, these approaches illustrate how computational antigen prioritization can support the development of individualized and multivalent circRNA vaccine constructs.

### 8.2 Engineering circRNAs to enhance stability and scalability

Following antigen selection, circRNA sequences must be engineered to achieve efficient translation, structural integrity, and reproducible production. Although the covalently closed-loop structure of circRNAs provides resistance to exonuclease-mediated degradation, achieving therapeutically relevant levels of antigen expression requires additional engineering strategies (Zhen et al., 2026). Since circRNAs lack the conventional 5′ cap and 3′ poly(A) tail, protein synthesis relies on cap-independent translation mechanisms. To address this limitation, researchers frequently incorporate IRESs and exploit m6A-mediated translation pathways to enhance protein production (Gao and Wu, 2025; Zhou et al., 2020). Optimization of coding sequences through codon engineering, incorporation of translation-enhancing motifs, and careful selection of spacer sequences can further improve antigen expression while maintaining structural integrity. Several circularization strategies have been developed to generate engineered circRNAs, each offering distinct advantages and limitations in terms of circularization efficiency, product fidelity, scalability, and suitability for therapeutic applications (Table 3).

**TABLE 3:** Comparison of major circRNA circularization strategies.

Circularization strategy	Principle	Major advantages	Major limitations	References
Chemical ligation	Chemical joining of the 5′ and 3′ termini of a linear RNA precursor	Can accommodate chemically modified RNA; does not require biological enzymes	Generally low efficiency; intermolecular ligation/oligomerization; less suitable for long RNA molecules	https://www.sciencedirect.com/science/article/pii/S2665906923000053
T4 DNA ligase-mediated ligation	Enzymatic ligation of RNA termini positioned by a complementary splint	Precise ligation; sequence-defined products; adaptable to different substrates	Requires splint optimization; affected by RNA secondary structure; intermolecular ligation can occur	https://www.cell.com/chem/abstract/S2451-9294(26)00011-2
T4 RNA ligase 1	Direct enzymatic joining of 5′-phosphate and 3′-OH termini	Relatively efficient; splint may not be required; can generate precise circular products	Sequence/terminal nucleotide preferences; intermolecular ligation; reduced efficiency with large RNAs	https://www.sciencedirect.com/science/chapter/bookseries/abs/pii/S1874604708602747
T4 RNA ligase 2	Enzymatic ligation, particularly efficient when termini are positioned within structured/dsRNA regions	Greater substrate flexibility; useful for structured RNA substrates; can generate precise junctions	Sensitive to RNA structure; intermolecular ligation; limited efficiency with very large RNAs	https://www.sciencedirect.com/science/chapter/bookseries/abs/pii/S1874604708602747
PIE—group I intron	Self-splicing of a permuted group I intron flanking the desired RNA sequence	Efficient production of larger circRNAs; applicable *in vitro* and *in vivo*; well established for therapeutic circRNA production	Intron/exon-derived sequences may remain; splicing by-products; sequence/structural constraints	https://www.nature.com/articles/s41467-018-05096-6
PIE—group II intron	Group II intron-mediated self-splicing and circularization	Can generate circRNAs with high sequence fidelity and can mimic endogenous circRNA sequences	Less extensively characterized than group I PIE systems	https://www.nature.com/articles/s41467-018-05096-6
Hairpin ribozyme	Ribozyme-mediated cleavage and ligation of RNA ends	High yields for relatively small circRNAs; good sequence homogeneity	Additional ribozyme-related sequences may remain; limited suitability for large RNAs	https://scholar.google.com/scholar?hl=en&as_sdt=0%2C5&q=Hairpin+ribozyme%09Ribozyme-mediated+cleavage+and+ligation+of+RNA+ends&btnG=
Tornado/tricRNA system	Ribozyme processing followed by RtcB-mediated RNA ligation	Efficient intracellular circularization; stable circRNA production; independent of canonical splicing	Residual intronic/ribozyme-derived sequence (“scar”) at the junction	https://www.sciencedirect.com/science/article/abs/pii/S1046202321001353

In parallel, considerable efforts have been directed toward improving the scalability of circRNA production. Current manufacturing approaches primarily rely on *in vitro* transcription followed by enzymatic or ribozyme-mediated circularization. Advances in self-splicing intron systems, permuted intron-exon technologies, and optimized ligation strategies have significantly increased circularization efficiency while reducing production costs (Wang L. et al., 2024; 2025). These improvements are critical for large-scale manufacturing, particularly if circRNA vaccines are to be implemented in personalized oncology settings where rapid production of patient-specific formulations may be required. The ability to generate highly stable circRNA constructs with reproducible quality represents a major step toward clinical translation and commercial viability.

### 8.3 Purification and quality control

The successful clinical application of circRNA vaccines depends not only on efficient production but also on rigorous purification and quality-control procedures. Following circularization, manufacturing reactions often contain residual linear RNA molecules, incomplete circularization products, double-stranded RNA contaminants, enzymes, and other reaction by-products that may affect vaccine performance or trigger unintended immune responses (Bahrulolum et al., 2026). Consequently, purification represents a critical step in ensuring the safety and efficacy of circRNA-based therapeutics.

Several analytical and purification methods have been developed to address these challenges. Techniques such as RNase R treatment, high-performance liquid chromatography (HPLC), gel electrophoresis, and capillary electrophoresis are frequently employed to enrich circular RNA populations and remove impurities (Abe et al., 2022; Cheng et al., 2024; Wesselhoeft et al., 2018; Zhang Q. et al., 2026). In addition, sequencing-based approaches and molecular characterization techniques are used to confirm sequence fidelity, circularization efficiency, and structural integrity (Ruan et al., 2023). RNase R treatment is particularly useful for degrading residual linear RNA while enriching the covalently closed circRNA fraction, whereas chromatographic approaches such as HPLC can further separate circRNAs from incompletely circularized products and other RNA species (Wang et al., 2026; Zheng et al., 2026). In addition, dsRNA contaminants generated during *in vitro* transcription can be reduced using chromatographic and cellulose-based purification approaches, thereby minimizing unwanted activation of innate immune responses (Liu J. et al., 2026). These methods allow researchers to evaluate not only the purity and stability of the circRNA but also its identity, structural integrity, and circularization efficiency. For therapeutic circRNA production, combining enzymatic treatment with orthogonal purification methods is important for achieving a highly purified product with consistent quality (Ugwu et al., 2026).

Quality control of purified circRNA requires assessment of identity, integrity, purity, circularization efficiency, stability, and functional activity (Moon et al., 2026). RNA identity and integrity can be evaluated using agarose gel electrophoresis or capillary electrophoresis, while resistance to RNase R digestion provides evidence for the presence of covalently closed RNA (Cieri et al., 2026; Elgohary et al., 2026). The back-splice junction can be confirmed by outward-facing reverse-transcription PCR followed by Sanger sequencing, providing sequence-level verification of circularization. Quantitative assessment of circularization efficiency and residual linear RNA can be further supported by electrophoretic or chromatographic analysis. In addition, dsRNA-specific assays can be used to monitor immunostimulatory RNA impurities, whereas stability studies under defined storage and handling conditions are required to evaluate product integrity over time. For therapeutic circRNA vaccines, functional quality control should ultimately include a potency assessment based on antigen expression or other relevant biological activity (Lee et al., 2026). Despite these advances, translating circRNA production from laboratory-scale workflows to Good Manufacturing Practice (GMP)-compatible manufacturing remains challenging. Large-scale production requires reproducible circularization, efficient removal of linear RNA and dsRNA contaminants, high recovery yields, and consistent product quality across batches. Gel-based purification is generally unsuitable for large-scale manufacturing, whereas chromatographic approaches offer greater scalability but may involve substantial processing costs, RNA losses, and optimization of separation conditions (Minkner et al., 2022). In addition, manufacturing processes must establish robust control of critical quality attributes, including RNA identity, purity, circularization efficiency, residual impurities, stability, and potency (Somara and Heidaran, 2026). Standardized analytical methods, validated purification processes, and well-defined release criteria will therefore be essential for establishing GMP-compliant manufacturing platforms for therapeutic circRNAs (Fallatah et al., 2026). Establishing standardized manufacturing and analytical protocols will be essential for regulatory approval and for ensuring batch-to-batch consistency as circRNA vaccines progress toward clinical development.

### 8.4 Delivery systems

Despite their enhanced stability, circRNA molecules require efficient delivery vehicles to overcome biological barriers and achieve effective intracellular antigen expression. Naked circRNAs are susceptible to limited cellular uptake and may fail to reach antigen-presenting cells in sufficient quantities to induce robust immune responses. Therefore, the development of suitable delivery systems has become a central aspect of circRNA vaccine research. The major delivery platforms investigated for circRNA vaccines differ considerably in their delivery efficiency, immunogenicity, manufacturing complexity, and translational potential (Table 4). Among currently available approaches, lipid nanoparticles (LNPs) represent the most advanced and widely adopted delivery platform. LNPs protect circRNAs from extracellular degradation, facilitate cellular uptake through endocytosis, and promote endosomal escape, thereby enhancing antigen expression within target cells (Das et al., 2025). The remarkable success of LNP-based mRNA vaccines during the COVID-19 pandemic has accelerated their adoption for circRNA delivery, and several preclinical studies have demonstrated efficient immune activation using LNP-encapsulated circRNA vaccines (Swingle et al., 2025).

**TABLE 4:** Comparison of delivery platforms for circRNA-based cancer vaccines.

Delivery platform	Advantages	Limitations	Relevance to circRNA vaccines
Lipid nanoparticles (LNPs)	Efficient intracellular delivery; protects circRNA from degradation; scalable formulation; clinically validated platform for RNA therapeutics	Potential innate immune activation; formulation and tissue-targeting challenges; possible toxicity at high doses	Most advanced and widely investigated platform for circRNA vaccine delivery
Viral vectors	High transduction efficiency; effective intracellular delivery; potential for tissue-specific targeting	Pre-existing immunity; limited cargo capacity for some vectors; manufacturing complexity; safety concerns	Potentially effective but comparatively less established for circRNA vaccine delivery
Polymeric nanoparticles	Tunable physicochemical properties; surface modification and targeting possible; potentially improved stability	Variable delivery efficiency; formulation complexity; possible polymer-associated toxicity	Promising alternative platform requiring further optimization
Exosome-based systems	Natural biocompatibility; intrinsic cellular uptake; potential for tissue targeting; low immunogenicity	Low and variable loading efficiency; isolation and characterization challenges; limited manufacturing scalability and standardization	Emerging platform with potential for targeted circRNA delivery

Beyond LNPs, researchers are actively investigating alternative delivery vehicles including polymeric nanoparticles, lipoplexes, extracellular vesicles, and biomimetic nanocarriers. Extracellular vesicles have attracted particular attention because of their natural biocompatibility, low immunogenicity, and inherent ability to mediate intercellular communication. Similarly, biomimetic delivery systems offer opportunities for tissue-specific targeting and enhanced accumulation within lymphoid organs, potentially improving vaccine efficacy while minimizing off-target effects. Although many of these technologies remain at the experimental stage, continued innovation in delivery science is expected to further enhance the therapeutic potential of circRNA-based cancer vaccines.

## 9 From preclinical development to clinical translation of circRNA-based cancer vaccines

The successful engineering of circRNA constructs and the development of efficient delivery platforms have paved the way for evaluating their therapeutic potential in cancer. Over the past few years, circRNA-based vaccines have rapidly evolved from conceptual RNA platforms into experimentally validated cancer immunotherapies. Their superior molecular stability, prolonged antigen expression, efficient cap-independent translation, and resistance to exonuclease-mediated degradation provide important advantages over conventional linear mRNA vaccines, thereby supporting sustained immune activation and durable antitumor responses (Deviatkin et al., 2023; Li et al., 2022; Wang et al., 2025; Zhang et al., 2025b; Zhen et al., 2026). These favorable characteristics have accelerated the progression of circRNA vaccines from proof-of-concept studies to extensive preclinical validation and, more recently, to early clinical development. Extensive preclinical investigations have consistently demonstrated that engineered circRNA vaccines induce potent antigen-specific immune responses across multiple tumor models. Sustained antigen expression promotes efficient dendritic-cell maturation, enhanced antigen presentation, robust CD8^+^ cytotoxic T-cell activation, increased CD4^+^ T-cell responses, elevated cytokine production, and the establishment of durable immunological memory (Huang D. et al., 2024; Wang F. et al., 2024; Zhang et al., 2025c). Collectively, these findings indicate that circRNA vaccines generate long-lasting cellular immunity, which is essential for effective therapeutic cancer vaccination.

A major milestone in the field was the demonstration that lipid nanoparticle (LNP)-encapsulated circRNA vaccines efficiently delivered tumor antigens *in vivo* and elicited potent innate and adaptive immune responses (Li et al., 2022). Furthermore, combining circRNA vaccination with adoptive T-cell therapy or immune checkpoint blockade resulted in superior therapeutic efficacy, highlighting the versatility of circRNA vaccines for combination cancer immunotherapy (Das et al., 2025). Building upon these encouraging findings, subsequent studies focused on optimizing vaccine design to further enhance translational efficiency and therapeutic performance. Improvements in IRES engineering, codon optimization, untranslated region design, and circularization strategies have substantially increased protein expression and vaccine potency (Zhang et al., 2025a; Zhang Z. et al., 2024). In parallel, advances in delivery technologies, including optimized lipid nanoparticles, lymph-node-targeting formulations, and lyophilized vaccine systems, have improved biodistribution, storage stability, and antigen presentation, thereby overcoming several practical limitations associated with RNA therapeutics (Cao et al., 2025; Li et al., 2025).

Recent investigations have also expanded the application of circRNA vaccines toward personalized cancer immunotherapy. circRNA vaccines encoding patient-specific neoantigens have demonstrated remarkable efficacy in generating highly specific cytotoxic T-cell responses while simultaneously remodeling the immunosuppressive TME (Xie et al., 2025). These personalized vaccine platforms have shown both preventive and therapeutic antitumor activity in preclinical hepatocellular carcinoma and melanoma models, supporting the feasibility of individualized circRNA vaccine strategies for precision oncology. The robust efficacy demonstrated in preclinical studies has facilitated the transition of circRNA-based therapeutics toward early clinical development. As shown in Table 2, circRNA vaccine development has progressed from preclinical antigen-delivery platforms to early-phase clinical evaluation, demonstrating the growing translational potential of this technology. Although no circRNA-based cancer vaccine has yet progressed to late-stage clinical trials, several first-in-human studies have recently been initiated. Notably, the ongoing Phase I clinical trial evaluating the CircFAM53B-219aa dendritic-cell vaccine in HER2-negative advanced breast cancer represents one of the earliest clinical applications of a circRNA-derived tumor antigen vaccine (Jiang et al., 2025). Additional early-phase studies investigating circRNA vaccine platforms for HPV-associated malignancies and advanced solid tumors further demonstrate the growing translational momentum of this technology (Zhang et al., 2025c). Beyond these clinical studies, the rapid expansion of the industrial landscape further illustrates the growing maturity of circRNA therapeutics. Biotechnology companies such as Orna Therapeutics, Sail Biomedicines, and CirCode Biomedicine have established dedicated circular RNA platforms, while strategic collaborations between biotechnology innovators and major pharmaceutical companies, including the partnership between Orna Therapeutics and Eli Lilly, reflect increasing commercial investment in circular RNA technologies and their potential application in oncology (Albert, 2022).

The transition from laboratory research to clinical implementation is further supported by advances in scalable circRNA manufacturing, purification technologies, and regulatory science. Efficient circularization strategies, robust purification methods to eliminate residual linear RNA and double-stranded RNA contaminants, and Good Manufacturing Practice (GMP)-compliant production processes are improving the feasibility of large-scale manufacturing (Buckland et al., 2024; Eshaghi et al., 2026). Concurrently, evolving regulatory frameworks established by agencies such as the FDA and EMA are providing guidance for product characterization, Chemistry, Manufacturing and Controls (CMC), analytical validation, and long-term safety evaluation, thereby laying the foundation for future clinical approval of circRNA-based therapeutics (Chen K. et al., 2025). Despite these encouraging advances, circRNA-based cancer vaccines remain at an early stage of clinical development. Most available evidence is derived from preclinical animal models, while human clinical data are currently limited to early-phase safety and feasibility studies. Comprehensive evaluation of long-term efficacy, optimization of personalized neoantigen selection, identification of predictive biomarkers, and validation in large patient cohorts will be essential before circRNA vaccines can be integrated into routine clinical oncology. Looking forward, continued advances in precision medicine, artificial intelligence-assisted neoantigen prediction, RNA engineering, and combination immunotherapy are expected to further accelerate the clinical translation of circRNA vaccines. Together with sustained collaboration among academia, biotechnology companies, pharmaceutical industries, regulatory agencies, and healthcare systems, these developments are anticipated to facilitate the commercialization of circRNA-based therapeutics and establish them as a next-generation platform for precision cancer immunotherapy.

## 10 Challenges and limitations

Despite remarkable advances in circRNA engineering, the clinical translation of circRNA based cancer vaccines is still at an early stage. Several biological, technical, and regulatory challenges must be addressed before these vaccines can become clinically established. Key unresolved issues include reproducible manufacturing, control of antigen expression and innate immune activation, efficient delivery, tumor heterogeneity, personalized vaccine production, limited clinical evidence, and regulatory standardization. One of the major challenges is achieving efficient and reproducible circRNA manufacturing (Cai et al., 2024). Although ribozyme mediated self-circularization has significantly improved circularization efficiency, current manufacturing workflows still generate residual linear RNA, double stranded RNA contaminants, and other reaction by-products (Lee et al., 2024). These impurities may alter the innate immune response, reduce translational efficiency, and introduce batch-to-batch variability, highlighting the need for standardized purification methods and robust quality control during large scale manufacturing (Chen H. et al., 2026). However, standardized critical quality attributes defining acceptable circularization efficiency, structural integrity, purity, and biological potency have yet to be established across different circRNA manufacturing platforms.

Another important challenge is balancing sustained antigen expression with controlled innate immune activation. CircRNAs rely on cap-independent translation, making protein production highly dependent on IRESs, RNA secondary structure, and sequence design (Chen R. et al., 2023; Zhang et al., 2025a). At the same time, excessive activation of innate immune sensors such as RIG-I, MDA5, and PKR may suppress translation and diminish vaccine efficacy (Okechukwu Paul-Chima et al., 2026). Thus, an important unresolved question is how to preserve the immunostimulatory properties of circRNAs without triggering excessive innate sensing that compromises antigen production. Achieving an appropriate balance between antigen persistence and immune activation will therefore be critical for vaccine optimization.

Delivery represents another major barrier to clinical translation. Lipid nanoparticles currently represent the most advanced delivery platform; however, they still face challenges related to tissue-specific targeting, endosomal escape, biodistribution, and potential inflammatory toxicity. Alternative carriers, including extracellular vesicles, biomimetic nanoparticles, and ligand modified delivery systems, show promise but require further optimization before clinical implementation (Wang et al., 2023). In particular, the optimal delivery strategy, biodistribution profile, and cellular targets required for efficient circRNA uptake by antigen-presenting cells *in vivo* remain insufficiently defined.

Tumor heterogeneity presents an additional challenge for circRNA-based vaccination (Huang S. et al., 2024). Tumours differ substantially in their antigenic composition, mutation burden, and immune microenvironment, allowing tumour cells to escape immune surveillance (Vitale et al., 2019). Consequently, vaccines targeting a single tumour associated antigen may provide limited efficacy across diverse patient populations (Reyes et al., 2017). Personalized circRNA vaccines encoding patient-specific neoantigens offer a potential strategy to address this limitation, but their clinical implementation may be constrained by the complexity, cost, and turnaround time of individualized neoantigen selection and vaccine production (Hussain et al., 2025).

Perhaps the most important limitation is the scarcity of clinical evidence. Most studies demonstrating the therapeutic efficacy of circRNA vaccines have been conducted in preclinical models, whereas clinical evidence remains limited. Consequently, important questions regarding long-term safety, optimal dosing, durability of immune responses, pharmacological behavior, and therapeutic efficacy remain insufficiently answered. In parallel, regulatory frameworks and standardized quality requirements specifically applicable to circRNA therapeutics remain under development, creating additional challenges for clinical translation and commercialization.

Collectively, these limitations indicate that successful translation of circRNA-based cancer vaccines will require advances beyond RNA engineering alone. Future studies must establish standardized manufacturing and quality-control criteria, define the determinants of efficient and controlled translation, optimize targeted delivery to relevant immune-cell populations, improve the reliability of neoantigen selection, and generate robust clinical evidence regarding safety, dosing, durability, and therapeutic efficacy. Addressing these unresolved questions will be essential for determining whether the promising preclinical properties of circRNA vaccines can translate into reproducible clinical benefit.

## 11 Future perspective

circRNA represents a highly promising platform for next-generation cancer vaccines, particularly due to the rapid advancements in RNA therapeutics. By optimizing IRES elements, enhancing m6A-mediated translation, refining codon optimization, and improving circularization strategies, these strategies may improve translation efficiency, extend antigen expression, and enable more precise control of immune activation (Fischer and Leung, 2017; Huang et al., 2022; Jiang et al., 2021; Lu et al., 2021; Zhou et al., 2020). Integration of next-generation sequencing, cancer genomics, and artificial intelligence could improve the identification of immunogenic neoantigens and facilitate personalized vaccine design. However, the clinical utility of individualized vaccines will depend on improving the speed, cost, and reproducibility of neoantigen selection and vaccine production (Blass and Ott, 2021). Another promising direction is the development of multifunctional circRNA constructs encoding multiple tumor antigens, immune-stimulatory cytokines, or co-stimulatory molecules. Such designs could broaden antitumor immune responses and potentially reduce immune escape associated with tumor antigen heterogeneity. Combining circRNA vaccines with established immunotherapeutic or tumor-directed approaches, including immune checkpoint blockade, CAR-T cell therapy, radiotherapy, or chemotherapy, may further enhance antitumor responses by addressing complementary mechanisms of immune suppression and tumor survival (Huang et al., 2026). Advances in targeted delivery will also be critical. Next-generation lipid nanoparticles, extracellular vesicles, and ligand-modified nanocarriers could improve circRNA delivery to dendritic cells and lymphoid tissues while reducing nonspecific exposure and toxicity. Future studies should prioritize tissue distribution, cellular targeting, endosomal escape, and dose optimization in relevant *in vivo* models. Ultimately, clinical translation will require scalable and reproducible manufacturing, standardized purification and quality-control criteria, and harmonized regulatory frameworks. Continued clinical evaluation will be essential for defining safety, optimal dosing, durability of immune responses, and therapeutic efficacy of circRNA vaccines (Makkar, 2025).

Taken together, the future of circRNA-based cancer vaccines will depend on integrating molecular engineering with precision oncology, targeted delivery, standardized manufacturing, and rigorous clinical evaluation. Rather than representing simply an alternative to linear RNA vaccines, circRNAs may provide a versatile framework for personalized and combination immunotherapy. Establishing whether their advantages in stability, antigen expression, and immune modulation translate into meaningful clinical benefit will ultimately determine their role in precision cancer treatment.
